# Supplementary material for: COVID-19 hospitalizations in Brazil’s Unified Health System (SUS)
Source: PLoS One. 2020 Dec 10;15(12):e0243126. doi: 10.1371/journal.pone.0243126 (PMC7728222; doi:10.1371/journal.pone.0243126)
Supplement: Attachment — Submitted filename: Response to reviewers.docx [file pone.0243126.s001.docx]

PONE-D-20-27175
COVID-19 hospitalizations in Brazil’s Unified Health System (SUS)

Dear Editor,

Thank you for your comments!

We are of the opinion that the considerations received from you and the reviewers have allowed us to present a more concise, clear and consistent version of the manuscript. Additionally, it is important to note that the whole text was revised by an English native speaker and professional translator.

Please find answers for all the points raised below.

We look forward to hearing from you regarding your final decision.

Best regards,

Margareth C. Portela

(on behalf of all the authors)

**EDITOR’S AND REVIEWERS’ COMMENTS AND AUTHOR’S ANSWERS**

***EDITOR***

*Thank you for submitting your manuscript to PLOS ONE. After careful consideration, we feel that it has merit but does not fully meet PLOS ONE’s publication criteria as it currently stands. Therefore, we invite you to submit a revised version of the manuscript that addresses the points raised during the review process.*

*Two reviewers have assessed the manuscript and provide discordant decisions. The main reasons for reject the paper is some concerns about study validity. Please, the new version should include specific information about study validity to be the manuscript considered to publication.*

**The concerns with the validity of the study aroused especially from the underreporting of comorbidities, but there were also the indications, by one of the two reviewers, of “lack of novelty and failed to provide new information regarding risk factors of in-hospital mortality”.**

**Our first point is that administrative data may be really important economic and prompt sources in observational studies in the Health Services Research field. The SUS Hospital Information System (SIH), as other administrative data worldwide, have been broadly employed in scientific studies, despite the inherent limitations. The SIH is the main source of information on hospital production in Brazil. It includes all hospitalizations in the Brazilian public system, responsible for covering 75% of the Brazilian population in the whole country. The anonymized SIH database is freely accessible in DATASUS website, and, to the best of our knowledge, our study is the first to use them to picture COVID-19 pandemic in Brazil. Even having some flow problems and not being available in real time, the data is mostly available in relatively short time. In the course of a pandemic in which evidence still needs to be accumulated, and Brazil has unquestionable importance as, at this point, the third country in number of cases and second in number of deaths, the role it can play is not negligible. At the same time, we are honest and explicitly recognize in the text the weaknesses of the data, arguing that they are supplanted by the contributions the study brings out.**

**Despite the efforts made in the last years to expand the number of fields for secondary diagnoses from one to nine in the SIH, they are not fulfilled. We truly have reasons to believe, based in previous studies and the experience as researchers, that there is a bad culture of clinical data underreporting in the country. If the study were based on the medical records in the hospitals, we would still have a lot of missing data. Hospitals with research activities often keep separate data for patients included in studies. The alternative of only be confident on primary data, however, would be unpractical and would neglect the capacity of secondary data provide useful information.**

**In our data, 78.3% of hospitalizations did not have a secondary diagnosis. It is likely that the COVID-19 scenario had even contributed for worse underreporting of comorbidities. In fact, data misrecording is expected in urgency contexts as the pandemic. Despite the problem, the death predictive capacity of model 1, the one which accounts for the clinical predictors of inpatient mortality, was 0.69, borderline to the range that is considered adequate (0.70-0.80).**

**We developed the study from the perspective of health services researchers and not clinicians. The level of hospital mortality is seen as an outcome that result not only from clinical aspects, but also the quality of healthcare. It was not our goal to necessarily provide new information on clinical risk factors of in-hospital mortality, but the available evidence was employed and tested in the modeling process. The focus was to compare COVID-19 hospital mortality adjusted by severity, expressing variations in quality of care – effectiveness and access inequalities – between hospital ownership and geographic areas.**

**Even considering the papers available, more evidence on care delivered in different countries seems important yet. The high volume of inpatients analyzed, severity adjusted strategies, focus on quality of care, and comparing geographical areas inequalities are the strong points.**

*Please ensure that your manuscript meets PLOS ONE's style requirements, including those for file naming. The PLOS ONE style templates can be found at* [*https://journals.plos.org/plosone/s/file?id=wjVg/PLOSOne_formatting_sample_main_body.pdf*](about:blank) *and* [*https://journals.plos.org/plosone/s/file?id=ba62/PLOSOne_formatting_sample_title_authors_affiliations.pdf*](about:blank)

**This was done.**

*We suggest you thoroughly copyedit your manuscript for language usage, spelling, and grammar. If you do not know anyone who can help you do this, you may wish to consider employing a professional scientific editing service.*

*Whilst you may use any professional scientific editing service of your choice, PLOS has partnered with both American Journal Experts (AJE) and Editage to provide discounted services to PLOS authors. Both organizations have experience helping authors meet PLOS guidelines and can provide language editing, translation, manuscript formatting, and figure formatting to ensure your manuscript meets our submission guidelines. To take advantage of our partnership with AJE, visit the AJE website (*[*http://learn.aje.com/plos/*](about:blank)*) for a 15% discount off AJE services. To take advantage of our partnership with Editage, visit the Editage website (*[*www.editage.com*](about:blank)*) and enter referral code PLOSEDIT for a 15% discount off Editage services.  If the PLOS editorial team finds any language issues in text that either AJE or Editage has edited, the service provider will re-edit the text for free.*

*Upon resubmission, please provide the following:*

- *The name of the colleague or the details of the professional service that edited your manuscript*
- *A copy of your manuscript showing your changes by either highlighting them or using track changes (uploaded as a *supporting information* file)*
- *A clean copy of the edited manuscript (uploaded as the new *manuscript* file)*

**This was done.**

**The English reviewer was Derrick Guy Phillips (Tel.: 55-21-99182-0989) – Union-registered translator since 1978 and Sworn Public Translator certified by the Board of Trade in Rio de Janeiro since March 2010. / M.A. – Master of Arts Degree ‘summa cum laude’ in Modern Languages & Philosophy (French, Spanish, Portuguese & Philosophy) from the University of St Andrews. Scotland (September 1969 through June 1973). / Post-Graduate Diploma in “International Marketing for Language Graduates” from the University of Central London, England (September 1977 through June 1978) – Group Award and Special Mention. / Translator and Director of Feedback Traduções Ltda. (Translation Agency), since 1978.**

*If you are reporting a retrospective study of medical records or archived samples, please ensure that you have discussed whether all data were fully anonymized before you accessed them.*

**The data obtained were already anonymized.**

*Please note that according to our submission guidelines (*[*http://journals.plos.org/plosone/s/submission-guidelines*](about:blank)*), outmoded terms and potentially stigmatizing labels should be changed to more current, acceptable terminology. For example: “Pardo” should be changed to “mixed race” (as appropriate).*

**This was done.**

*In your Data Availability statement, you have not specified where the minimal data set underlying the results described in your manuscript can be found. PLOS defines a study's minimal data set as the underlying data used to reach the conclusions drawn in the manuscript and any additional data required to replicate the reported study findings in their entirety. All PLOS journals require that the minimal data set be made fully available. For more information about our data policy, please see* [*http://journals.plos.org/plosone/s/data-availability*](about:blank)*.*

*Upon re-submitting your revised manuscript, please upload your study’s minimal underlying data set as either Supporting Information files or to a stable, public repository and include the relevant URLs, DOIs, or accession numbers within your revised cover letter. For a list of acceptable repositories, please see* [*http://journals.plos.org/plosone/s/data-availability#loc-recommended-repositories*](about:blank#loc-recommended-repositories)*. Any potentially identifying patient information must be fully anonymized.*

*Important: If there are ethical or legal restrictions to sharing your data publicly, please explain these restrictions in detail. Please see our guidelines for more information on what we consider unacceptable restrictions to publicly sharing data:* [*http://journals.plos.org/plosone/s/data-availability#loc-unacceptable-data-access-restrictions*](about:blank#loc-unacceptable-data-access-restrictions)*. Note that it is not acceptable for the authors to be the sole named individuals responsible for ensuring data access.*

*We will update your Data Availability statement to reflect the information you provide in your cover letter.*

**OK. We are making the data used in the study available. The data is open access, as described in the manuscript.**

***REVIEWERS***

1. Is the manuscript technically sound, and do the data support the conclusions?

The manuscript must describe a technically sound piece of scientific research with data that supports the conclusions. Experiments must have been conducted rigorously, with appropriate controls, replication, and sample sizes. The conclusions must be drawn appropriately based on the data presented.

Reviewer #1: No

Reviewer #2: Yes

**As we have already indicated in this letter and in the manuscript, we acknowledge that the study has some limitations. However, we are also convinced that it provides a good overview of COVID-19 hospitalizations in the Brazilian Unified Health System in the first four months of the pandemic in Brazil and highlights the huge variation in in-hospital mortality, associated with social and healthcare quality inequities throughout the country. We maintain that our research is sound and the data support all conclusions provided. The study is replicable.**

2. Has the statistical analysis been performed appropriately and rigorously?

Reviewer #1: No

Reviewer #2: Yes

**We believe that we used the best statistical approach available for the research question at hand. We were interested in analyzing factors associated with in-hospital mortality due to Covid-19 and applied variables commonly used in the health service research literature to address this type of question. Furthermore, we were careful to use appropriate modelling to account for the violation of the assumption of independence among observations in the same hospital.**

3. Have the authors made all data underlying the findings in their manuscript fully available?

The [PLOS Data policy](about:blank#sharing) requires authors to make all data underlying the findings described in their manuscript fully available without restriction, with rare exception (please refer to the Data Availability Statement in the manuscript PDF file). The data should be provided as part of the manuscript or its supporting information, or deposited to a public repository. For example, in addition to summary statistics, the data points behind means, medians and variance measures should be available. If there are restrictions on publicly sharing data—e.g. participant privacy or use of data from a third party—those must be specified.

Reviewer #1: Yes

Reviewer #2: Yes

**OK.**

4. Is the manuscript presented in an intelligible fashion and written in standard English?

PLOS ONE does not copyedit accepted manuscripts, so the language in submitted articles must be clear, correct, and unambiguous. Any typographical or grammatical errors should be corrected at revision, so please note any specific errors here.

Reviewer #1: No

Reviewer #2: Yes

**We are providing a new version of the manuscript revised by an English native speaker and professional translator.**

5. Review Comments to the Author

Please use the space provided to explain your answers to the questions above. You may also include additional comments for the author, including concerns about dual publication, research ethics, or publication ethics. (Please upload your review as an attachment if it exceeds 20,000 characters)

*Reviewer #1: It's a large study covering Unified Health System in the COVID-19 pandemic. But I feel it's lack of novelty and failed to provide new information regarding risk factors of in-hospital mortality in patients with COVID-19. You mentioned that 78.3% of hospitalizations did not have any secondary diagnoses, which made me concern that there might be misdocumentation affecting the validity of the data. Also, it might be better to consider length of stay and ICU use as outcomes.*

**To the best of our knowledge, this is the first study using data from the SUS Hospital Information System, an administrative database which covers, nationally, all hospitalizations that take place in the SUS, that provides healthcare to 75% of the Brazilian population. We were able to analyze 89,405 hospitalizations across the country in the first four months of the COVID-19 pandemic in Brazil. Considering the importance of Brazil in the global COVID-19 pandemic scene, we would probably be making a bigger mistake if we neglected information which can be extracted from the database, and would have no better source, especially if swiftness and economic criteria were applied.**

**Despite the underreporting of comorbidities, the strong gradient observed for age in all models, which has even increased along the inclusion of the blocks of variables, seems to indicate age as a more reliable indicator to predict in-hospital death in the context.**

**Such analyses are needed to shine a light on in-hospital mortality and associated factors. Although much has been discussed in the media and in the academic output about comorbidities such as hypertension, obesity and diabetes, we found some novel results such as: the surprisingly higher chance of death when the length of stay was short (less than 24 hours), probably reflecting access barriers; higher odds ratio adjusted by severity in some areas, indicating effectiveness variability; the in-hospital mortality risk related to transference to another city for healthcare; specific differences among hospitals with different kinds of ownership.**

**Also, it is true that the important race gradient (higher mortality among blacks, followed by mixed race people) had already been shown in other studies, but it is likely to reflect marked social inequities that need to be exposed and addressed by policy makers. Accumulating evidence and understanding how COVID-19 outcomes are affected by other factors beyond clinical factors is the overriding goal.**

**With regard to the comment about the inclusion of length of stay (LOS) and ICU use as explanatory variables, we justify our choice underlining our health services researchers’ perspective. Both variables used are healthcare processing variables. ICU use ends up reflecting, to some extent, clinical severity, beyond the comorbidity data itself. LOS, in turn, seems to reflect healthcare access and quality. Our study design and analyses were not oriented towards detecting causality relations in which cause precedes effect. By including both variables, we sought to identify simply how the risk of in-hospital mortality varies vis-à-vis distinct standards of hospital stay duration and ICU use or not.**

**A data quality gap is to be expected in all sources of information, even medical records. The emergency room, ICU and wards were operating in crisis mode and under dramatic conditions. Based on the abovementioned points, we believe that all possible information should be obtained and examined to draw a more complete picture of the care provided, in various contexts and countries.**

Reviewer #2: The theme chosen for the study was of great relevance. The Title and abstract were adequate and consistent.

The introduction presented fundamental characteristics in its composition. As an example, I point out the description of the unique health system, peculiar to Brazil and necessary for the understanding of the study. I advise that the text between lines 77 to 99 could be summarized.
The objectives were clear. As a suggestion, I would include the last sentence after the objectives (lines 116 to 118) as a strong point of the work and not at the end of this paragraph.
The methods have been well described. I advise you to describe the Charlson and Elixhauser indices in this topic and not in the results. The results reported are consistent and very important topics. We advise you to keep the discussion more concise, valuing the most important results.

**Thank you for your careful review of our manuscript and comments about the adequacy of our title, abstract, objectives and results. We think that we attended all your recommendations.**

**We summarized the text between lines 77 and 99 of the previous version to nine lines (73-81) in the new version.**

**We also accepted your suggestion transferring the last sentence after the objectives to the end of the Discussion section, as a strong point of the work.**

**In fact, the descriptions and references of the Charlson and Elixhauser indices were already in the Methods section. We have highlighted them in the file with track changes, and added a few more explanations to make the text clearer. No explanations about them were left in the results.**

**We have worked on the Discussion and made it shorter by emphasizing the results that we considered most important.**
